# Guidelines for the diagnosis and management of cystathionine beta-synthase deficiency

**DOI:** 10.1007/s10545-016-9979-0

**Published:** 2016-10-24

**Authors:** Andrew A. M. Morris, Viktor Kožich, Saikat Santra, Generoso Andria, Tawfeg I. M. Ben-Omran, Anupam B. Chakrapani, Ellen Crushell, Mick J. Henderson, Michel Hochuli, Martina Huemer, Miriam C. H. Janssen, Francois Maillot, Philip D. Mayne, Jenny McNulty, Tara M. Morrison, Helene Ogier, Siobhan O’Sullivan, Markéta Pavlíková, Isabel Tavares de Almeida, Allyson Terry, Sufin Yap, Henk J. Blom, Kimberly A. Chapman

**Affiliations:** 1 Institute of Human Development, University of Manchester, Manchester, UK; 2 Willink Unit, Manchester Centre for Genomic Medicine, Central Manchester University Hospitals, St Mary’s Hospital, Oxford Road, Manchester, M13 9WL UK; 3 Institute of Inherited Metabolic Disorders, Charles University in Prague-First Faculty of Medicine and General University Hospital in Prague, Prague, Czech Republic; 4 Clinical IMD, Birmingham Children’s Hospital, Birmingham, UK; 5 Department of translational medicine, Federico II University, Naples, Italy; 6 Department of Pediatrics, Hamad Medical Corporation, Doha, Qatar; 7 Department of Metabolic Medicine, Great Ormond Street Hospital, London, UK; 8 National Centre for Inherited Metabolic Disorders, Temple Street Children’s University Hospital, Dublin, Ireland; 9 Biochemical Genetics, St James’ University Hospital, Leeds, UK; 10 Division of Endocrinology, Diabetes and Clinical Nutrition, University Hospital Zürich, Zurich, Switzerland; 11 Division of Metabolism and Children’s Research Center, University Children’s Hospital Zürich, Zurich, Switzerland; 12 Rare Disease Initiative Zürich, University of Zürich, Zurich, Switzerland; 13 Dept. of Paediatrics, Landeskrankenhaus Bregenz, Bregenz, Austria; 14 Department of Internal medicine, Radboud University Medical Center, Nijmegen, Netherlands; 15 CHRU de Tours, Université François Rabelais, Tours, France; 16 Newborn Bloodspot Screening Laboratory, Temple Street Children’s University Hospital, Dublin, Ireland; 17 HCU Network, Baulkham Hills, Australia; 18 Service de Neurologie Pédiatrique et des Maladies Métaboliques, Hôpital Robert Debré, Paris, France; 19 Royal Belfast Hospital for Sick Children, Belfast, UK; 20 Metabolism & Genetics Group, Faculty of Pharmacy at University of Lisboa, Lisboa, Portugal; 21 Dietetic Department, Alder Hey Hospital, Liverpool, UK; 22 Dept of Inherited Metabolic Diseases, Sheffield Children’s Hospital, Sheffield, UK; 23 Laboratory of Clinical Biochemistry and Metabolism, Department of General Pediatrics, Adolescent Medicine and Neonatology, University Medical Centre Freiburg, Freiburg im Breisgau, Germany; 24 Division of Genetic and Metabolism, Children’s National Health System, Washington, DC USA

## Abstract

Cystathionine beta-synthase (CBS) deficiency is a rare inherited disorder in the methionine catabolic pathway, in which the impaired synthesis of cystathionine leads to accumulation of homocysteine. Patients can present to many different specialists and diagnosis is often delayed. Severely affected patients usually present in childhood with ectopia lentis, learning difficulties and skeletal abnormalities. These patients generally require treatment with a low-methionine diet and/or betaine. In contrast, mildly affected patients are likely to present as adults with thromboembolism and to respond to treatment with pyridoxine. In this article, we present recommendations for the diagnosis and management of CBS deficiency, based on a systematic review of the literature. Unfortunately, the quality of the evidence is poor, as it often is for rare diseases. We strongly recommend measuring the plasma total homocysteine concentrations in any patient whose clinical features suggest the diagnosis. Our recommendations may help to standardise testing for pyridoxine responsiveness. Current evidence suggests that patients are unlikely to develop complications if the plasma total homocysteine concentration is maintained below 120 μmol/L. Nevertheless, we recommend keeping the concentration below 100 μmol/L because levels fluctuate and the complications associated with high levels are so serious.

## Introduction

Cystathionine beta-synthase (CBS) deficiency is a rare inherited disorder, also known as classical homocystinuria (OMIM 236200). Homocysteine (Hcy) is a non-structural amino acid (AA) that is formed in the catabolic pathway for the essential AA, methionine (Met). CBS deficiency impairs the conversion of Hcy to cystathionine and leads to its accumulation.

Patients with CBS deficiency show a wide spectrum of severity and age at presentation. Some patients have a severe childhood-onset multisystem disease, whilst others are asymptomatic into adulthood. The main clinical features are dislocation of the optic lenses, osteoporosis and a ‘marfanoid’ habitus, learning difficulties and a predisposition to thromboembolism. Other causes of hyperhomocysteinemia include inborn errors of Hcy remethylation, vitamin deficiencies (especially B_12_), renal insufficiency and medication.

### Prevalence

CBS deficiency occurs worldwide but the prevalence varies widely depending on ethnicity and the method of ascertainment. The true population frequency is unknown with estimates ranging from 1:1800 to 1:900,000 based on birth incidence of patients detected by newborn screening and/or estimates from clinically ascertained patients (Mudd et al 1995; Zschocke et al 2009; Gan-Schreier et al 2010). The highest incidence has been reported in Qatar (1:1800), where there is a high rate of consanguinity and a founder effect, with a carrier frequency of approximately 2 % (Zschocke et al 2009; Gan-Schreier et al 2010). Several molecular epidemiological studies in European populations analysed frequency of heterozygotes for selected mutations with subsequent calculation of the expected population frequency of homozygotes (Gaustadnes et al 1999; Refsum et al 2004a, b; Janosik et al 2009). These studies indicate that the prevalence may have been underestimated, however, the data also suggest that some homozygotes for mutation c.833 T > C may be asymptomatic (Skovby et al 2010).

### Biochemistry

**Fig. 1:**
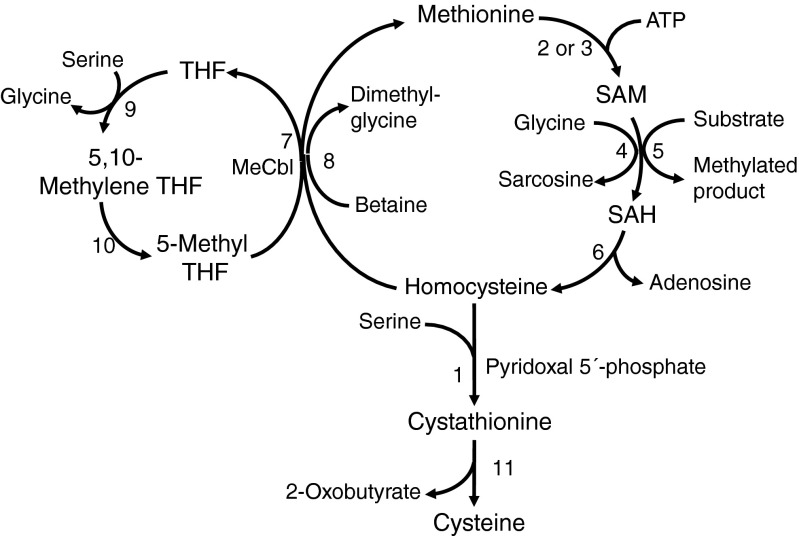
Pathways of methionine metabolism. SAM, S-adenosylmethionine; SAH, S-adenosylhomocysteine; THF, tetrahydrofolate; MeCbl, methylcobalamin. 1, cystathionine beta-synthase; 2, methionine adenosyltransferase I/III; 3, methionine adenosyltransferase II; 4, glycine N-methyltransferase; 5, numerous methyltransferases; 6, S-adenosylhomocysteine hydrolase; 7, methionine synthase; 8, betaine-homocysteine methyltransferase; 9, Serine hydroxymethyltransferase; 10, methylenetetrahydrofolate reductase; 11, cystathionine gamma-lyase

The pathways of Met metabolism are shown in Fig. 1. Met is converted to Hcy via S-adenosylmethionine (SAM) and S-adenosylhomocysteine (SAH), releasing a methyl group which is used in numerous methylation reactions. Glycine N-methyltransferase acts on any excess SAM that is not used in these reactions. Hcy can be converted back to Met by the remethylation pathway. The methyl-group donor can either be 5-methyltetrahydrofolate (catalysed by methionine synthase, with methylcobalamin as a cofactor) or betaine (especially in patients treated with this drug). Alternatively, Hcy is irreversibly metabolised to cysteine by the transsulfuration pathway. This starts with condensation of Hcy and serine to form cystathionine, catalysed by CBS. Cystathionine is subsequently cleaved by cystathionine γ-lyase to form cysteine and 2-oxobutyrate. Cysteine can be further converted to taurine or to inorganic sulfate via hydrogen sulfide.

CBS is expressed predominantly in liver, pancreas, kidney and brain. Its catalytic domain binds heme and the cofactor pyridoxal 5′phosphate, in addition to its substrates; the regulatory domain binds SAM as an allosteric activator.

**Fig. 2:**
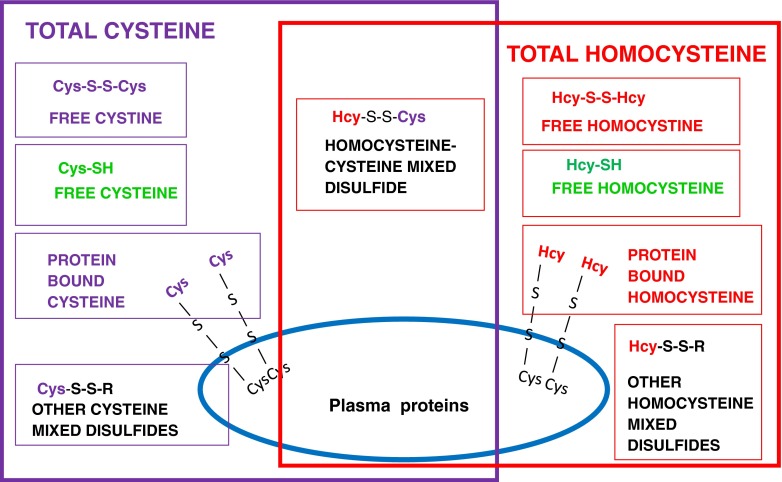
Various forms of aminothiols in plasma. Hcy-SH, free homocysteine; Cys-SH, free cysteine; Hcy-S-S-Hcy, free homocystine; Cys-S-S-Cys, free cystine; Hcy-S-S-Cys, mixed disulfide; Hcy-S-S-R and Cys-S-S-R, homocysteine and cysteine bound to other thiols

Hcy contains a thiol (–SH) group that reacts readily with other thiol groups. Hcy is, therefore, present in plasma in a variety of chemical forms (Fig. 2). These include the free reduced form (1 %), disulfides (homocystine and mixed disulfides with cysteine or other thiols, in total about 30 %) and protein-bound species (about 70 %) (Fowler and Jakobs 1998; Rasmussen and Moller 2000). The sum of the free and all bound Hcy species is defined as total homocysteine (tHcy). Cysteine also contains a thiol group and is present in different reduced and oxidised forms including mixed disulfides and protein-bound species. The binding capacity of plasma proteins is limited and grossly elevated concentrations of homocysteine lead to a decrease in plasma total cysteine. In CBS deficiency, diminished cysteine production via the transsulfuration pathway may also contribute to low cysteine concentrations.

### Pathogenesis

The pathophysiology of CBS deficiency is not fully understood. As well as the accumulation of Hcy, the defect leads to increased concentrations of SAH, enhanced remethylation to methionine, and depletion of cystathionine and cysteine. Raised Hcy concentrations modify sulfhydryl groups on proteins and interfere with the cross-linking of sulfhydryl groups in proteins such as elastin; this is thought to cause the lens dislocation and skeletal abnormalities. Raised Hcy concentrations also alter intracellular signaling and cause endoplasmic reticulum stress with endothelial dysfunction (Schienle et al 1995; Lai and Kan 2015); these mechanisms together with impaired thrombolysis may be responsible for thromboembolism and vascular disease (Schienle et al 1994). Increased SAH impairs methylation reactions and decreased cystathionine and cysteine are associated with apoptosis, oxidative stress and alterations of structural proteins such as fibrillin, which may contribute to connective tissue abnormalities. Altered synthesis of hydrogen sulfide may also contribute to the pathophysiology.

## Methodology and objectives

### Aims

This guideline aims to be a practical guide to the recognition, diagnosis and management of CBS deficiency. It is designed to be used by healthcare professionals who encounter this condition, including paediatricians, physicians, geneticists, biochemists, dietitians and psychologists. The guideline aims to consider all patients with CBS deficiency, though some sections will only apply to subgroups. Unfortunately, for many issues there is a lack of high quality evidence and practice varies considerably. Even for these issues, standardisation may bring advantages (e.g. for testing pyridoxine responsiveness).

### Guideline development

The guideline was written as part of the European network and registry for homocystinurias and methylation defects (E-HOD). A Guideline Development Group (GDG) was convened, including paediatricians, adult physicians, dietitians, biochemists, a clinical geneticist and a statistician. At the first meeting, in November 2013, methodology was agreed to ensure standardised literature evaluation, the key questions were decided and working groups were established to focus on these topics. Subsequently, GDG members undertook a systematic literature review. Statements and supporting evidence were prepared and discussed with the full group at two further meetings, in July 2014 and April 2015. Further revision was made following comments from a patient support group representative (Tara Morrison, Director and Chair of HCU Network Australia) and two highly renowned external reviewers (James V. Leonard and Bridget Wilcken).

### Systematic literature review and evidence grading

The methodology used for collecting the evidence base for this guideline is essentially that used by the Scottish Intercollegiate Guideline Network (SIGN, http://www.sign.ac.uk). A systematic literature review on CBS deficiency from the time of description until December 2013 was carried out, mainly using Pubmed; literature published after 2013 was added as needed. Relevant papers were each evaluated by a minimum of two GDG members. Evidence levels were classified in accordance with the SIGN methodology (Table 1) and recommendations given in the guideline were graded depending on their level of evidence (Table 2).

**Table 1:** SIGN methodology

Evidence level	Criteria
1++	High quality meta-analyses, systematic reviews of randomised control trials (RCTs), or RCTs with a very low risk of bias.
1+	Well conducted meta-analyses, systematic reviews of RCTs, or RCTs with a low risk of bias.
1-	Meta-analyses, systematic reviews or RCTs, or RCTs with a high risk of bias.
2++	High quality systematic reviews of case-control or cohort studies or high quality case control or cohort studies with a very low risk of confounding bias, or chance and a high probability that the relationship is causal.
2+	Well conducted case-control or cohort studies with a low risk of confounding, bias, or chance and a moderate probability that the relationship is causal.
2-	Case-control or cohort studies with a high risk of confounding, bias, or chance and a significant risk that the relationship is not causal.
3	Non-analytic studies, e.g. case reports, case series.
4	Expert opinion.

**Table 2:** Grading according to SIGN

Grade of recommendation	Criteria
A	If level 1 evidence was found (never in this study)
B	If level 2 evidence was found
C	If level 3 evidence was found (mainly non-analytical studies such as case reports and case series)
D	If level 4 evidence was found (mainly expert opinion)

## Recommendations

Recommendations are organised as 41 separate statements. In the following sections, each statement (in italics) precedes the explanation and data used to craft the statement.

### Clinical presentation

#### Clinical features (statement #1: grade of recommendation B)

*There is a wide spectrum of severity*, *from individuals who are currently asymptomatic to those with severe multi-system disease*, *with a wide range of ages at presentation. The phenotype broadly relates to pyridoxine-responsiveness. Four main organs*/*systems can be involved*:

- ***EYE***: *ectopia lentis and*/*or severe myopia*
- ***Skeleton***: *excessive height and length of the limbs* (‘*marfanoid*’ *habitus*), *osteoporosis and bone deformities*, *such as pectus excavatum or carinatum*, *genu valgum and scoliosis*
- ***Central nervous system***: *developmental delay*/*intellectual disability*, *seizures*, *psychiatric and behavioural problems and extrapyramidal signs*
- ***Vascular system***: *thromboembolism*

Patients with CBS deficiency vary markedly in their symptoms, age of onset and rate of progression of clinical signs (Schimke et al 1965; Mudd et al 1985; Picker and Levy 1993; de Franchis et al 1998). The severe end of the spectrum, with multi-system disease, was described in early reports (Carson et al 1965; Schimke et al 1965). Subsequently, connective tissue, neuro-psychiatric and vascular presentations have been recognised (Kelly et al 2003; Linnebank et al 2003; Magner et al 2011; Zaidi et al 2012; Karaca et al 2014).

Pyridoxine-responsive patients generally have a milder phenotype and a later onset than the pyridoxine-unresponsive ones (Mudd et al 1985; Abbott et al 1987). At the mildest end of the spectrum, there is a large group of patients who are extremely sensitive to pyridoxine, often achieving normal tHcy levels on small doses. It is likely that many of these patients remain asymptomatic throughout life, especially those homozygous for the p.I278T mutation (Skovby et al 2010). Others present as adults with thromboembolism; a few have ectopia lentis but most have no other complications. For these patients, the most relevant sections of the guideline are 3A, 3B1-7, 3D, 3I3, 3 J and 3 K. The diagnosis is often missed in these patients and the group has been under-represented in most series, including the classical description of the natural history (Mudd et al 1985). For this reason, it is unknown what proportion of pyridoxine-responsive patients develop complications.

Eye: ectopia lentis (dislocation of the lenses) is the ocular hallmark of the disease. Without treatment, it usually occurs during childhood, at least in pyridoxine unresponsive patients. It may be preceded by progressive myopia (Mudd et al 1985; Picker and Levy 1993).

Skeleton: patients tend to have excessive height and limb length (dolichostenomelia and arachnodactyly) and are prone to osteoporosis, at least after childhood. Various bone deformities and abnormal X-ray findings may also be present (Brenton 1977).

Central nervous system: intellectual, psychiatric and behavioural problems are all common, especially in pyridoxine unresponsive patients (Abbott et al 1987; El Bashir et al 2015). Without treatment, approaching 90 % pyridoxine unresponsive patients have learning difficulties (Mudd et al 1985). Seizures and extrapyramidal signs are also quite frequent features.

Vascular system: thromboembolism is the major cause of morbidity and early death. Venous thrombosis is more common than arterial but it can affect any part of the body (Mudd 1985; Magner et al 2011; Karaca et al 2014).

#### Common presentations (statement #2: grade of recommendation B-C)

*Children are more likely to present with developmental delay and ectopia lentis. Adults are more likely to be diagnosed following vascular events. Most manifestations*, *however*, *can occur at almost any age*.

Patients with CBS deficiency can present to a wide range of specialists, including pediatricians, ophthalmologists, haematologists, neurologists, psychiatrists, orthopedic surgeons, cardiologists, vascular specialists and clinical geneticists (Mudd et al 1985; Cruysberg et al 1996). Ocular findings and developmental delay are important clinical signs in children that may lead doctors to suspect CBS deficiency. Ectopia lentis is particularly suggestive and should alert any ophthalmologist to this diagnosis. The diagnosis should also be considered if there is severe or rapidly progressing lenticular myopia. Vascular complications, such as cerebral venous sinus thrombosis, can occur in childhood (Mudd et al 1985; Karaca et al 2014) but vascular presentations without other features are much more frequent in adulthood (Mudd 1985; Kelly et al 2003; Linnebank et al 2003; Magner et al 2011).

#### Differential diagnosis (statement #3: grade of recommendation B)

*The differential diagnosis of the presenting features is very broad and includes Marfan syndrome and prothrombotic disorders*.

Severely affected patients with CBS deficiency share some clinical features with Marfan syndrome, including tall slender body habitus, arachnodactyly, lens dislocation and myopia (Schimke et al 1965). Other genetic conditions associated with non-traumatic lens dislocation include Weill Marchesani syndrome, Ehlers Danlos syndrome and sulfite oxidase deficiency (Sadiq and Vanderveen 2013). To ensure that a treatable diagnosis is not missed, CBS deficiency should be excluded in all cases by measuring plasma tHcy.

There are a wide range of causes for thromboembolism, psychiatric problems and intellectual disability. CBS deficiency should be considered in the differential diagnosis, even if the patient shows no other clinical features of classical homocystinuria (Cruysberg et al 1996).

### Diagnosis

#### Biochemical diagnosis (statement #4: grade of recommendation C)

*Plasma total homocysteine* (*tHcy*) *should be the frontline test for diagnosis of CBS deficiency. Plasma free homocystine* (*fHcy*) *only becomes detectable at tHcy concentrations above approximately 50-60 μmol*/*L*; *its measurement is not recommended because of its low sensitivity and reproducibility*, *and the demanding pre-analytical requirements. In untreated patients with CBS deficiency*, *tHcy concentrations are usually above 100 μmol*/*L but they may be lower. The diagnosis is very likely if elevated tHcy is accompanied by high or borderline high plasma Met concentrations and further supported if sensitive methods demonstrate low plasma cystathionine concentrations with an increased Met-to-cystathionine ratio*.

CBS deficiency is characterised biochemically by an accumulation of Hcy, decreased synthesis of cystathionine and cysteine and usually by increased Met (Orendac et al 2003; Stabler et al 2013). However, the classical pattern is not always observed in all variants of CBS deficiency (Stabler et al 2013).

Two indices of plasma homocysteine levels have been widely used, the free homocystine (fHcy)—the concentration of non-protein-bound disulfide homocystine—and the total homocysteine (tHcy)—the sum of all free and bound homocysteine species after treating plasma with a reducing agent (see Fig. 2 for the different Hcy pools). Measurement of fHcy has been possible since the 1960s, whereas tHcy has only been widely available since the late 1990s. The plasma fHcy determined by an AA analyser is an insensitive marker of CBS deficiency as it only becomes detectable once tHcy exceeds approximately 50-60 μmol/L (Moat et al 1999; see below for a larger study). In addition, this test is unreliable because the fHcy continues to bind to plasma proteins in vitro unless rapid deproteinization is carried out and meticulous pre-analytical measures have been observed. Thus, determination of plasma fHcy is no longer recommended for diagnosis or monitoring. Measurement of urinary disulfide homocystine is even less sensitive as this only becomes detectable once plasma tHcy exceeds approximately 150 μmol/L (Moat et al 1999).

**Fig. 3:**
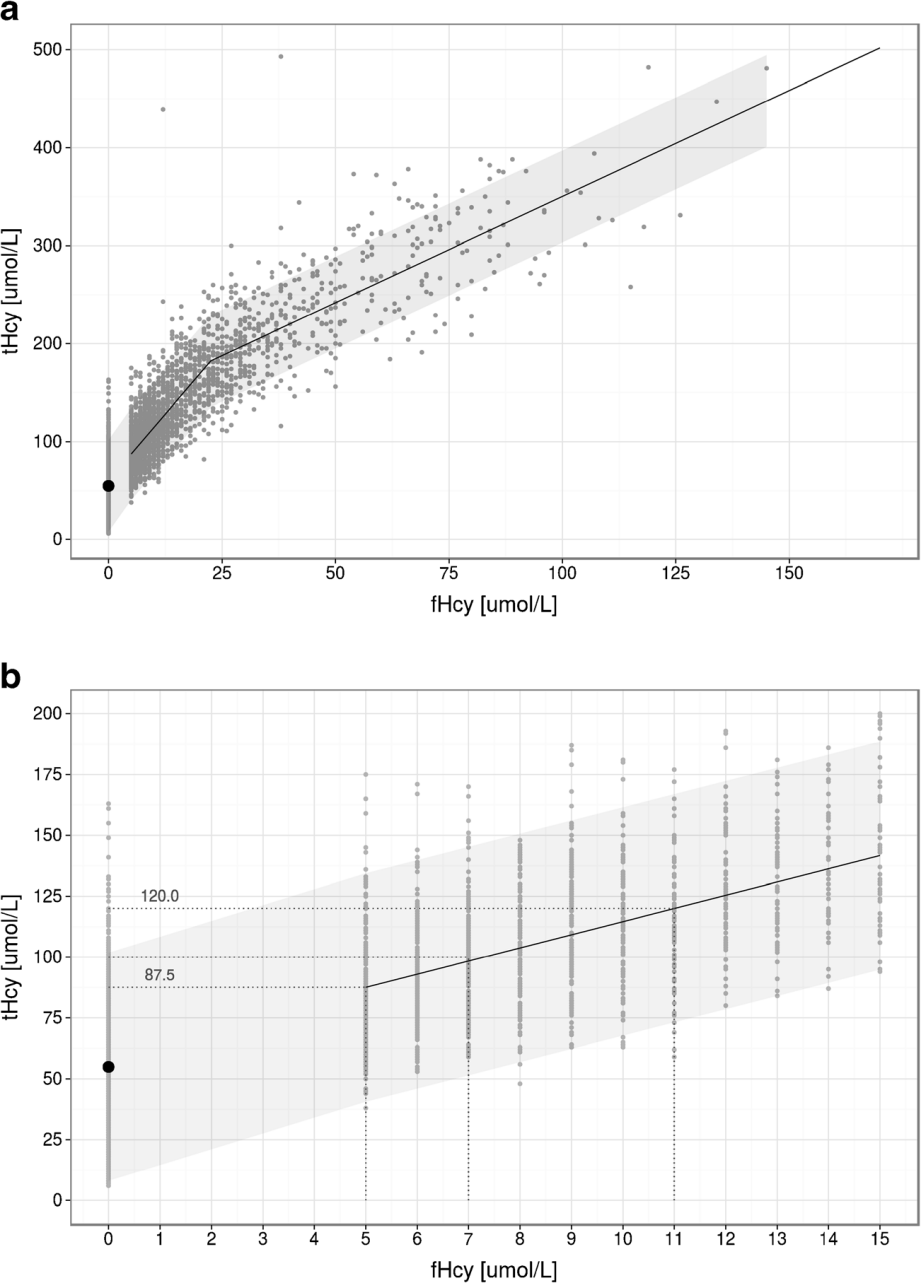
Relationship between plasma total homocysteine and free homocystine. Figure 3a shows the simultaneous tHcy and fHcy measurements in 3522 plasma samples collected from 46 Irish patients with CBS deficiency. Blood samples were obtained at the time of routine clinic visits. Plasma was separated within 15 minutes after collection and a 150 μL aliquot of plasma was immediately deproteinised by addition of 15 μL of 35 % sulphosalicylic acid; fHcy was measured by ion-exchange chromatography with ninhydrin detection. tHcy was measured on the aliquot of neat plasma by ion-exchange chromatography with ninhydrin detection following incubation with 2.5 % dithiothreitol. Segmented linear regression analysis was used because fHcy could not be quantified accurately below 5 μmol/L. The black point shows the model’s estimate of the tHcy concentration at which fHcy will start to be present. The shadowing around the regression line shows the 90 % confidence band within which 90 % tHcy values lie for a given fHcy value. The section of the graph for fHcy <15 μmol/L is expanded in Fig. 3b

Figure 3 shows the relationship between plasma tHcy and fHcy in a longitudinal study of 46 patients from a single centre. In plasma samples with fHcy below the detection limit, the tHcy concentration varied widely, with 5^th^ and 95^th^ centiles of 17 and 97 μmol/L, respectively. Segmented linear regression analysis indicates that one would expect free homocystine to be present when the tHcy concentration is above approximately 55 μmol/L. A fHcy concentration of 5 μmol/L corresponds to tHcy concentration of approximately 88 μmol/L; below this concentration, many amino acid analysers cannot quantify fHcy.

In untreated patients with CBS deficiency, plasma tHcy exceeds the upper limit of the reference range (which is generally between 10 and 15 μmol/L, though it varies with age and method of analysis); tHcy concentrations are typically above 100 μmol/L but considerably lower concentrations may be found in patients with mild variants of CBS deficiency (Kozich and Kraus 2001; Refsum et al 2004a, b; Bermudez et al 2006; Stabler et al 2013). The median tHcy in a cohort of 25 CBS deficient patients was 125 μmol/L with a range of 16-281 (Stabler et al 2013). Indeed, plasma tHcy concentrations may be normal in pyridoxine-responsive patients if they are receiving even low doses of pyridoxine in vitamins and food supplements.

Further biochemical support for CBS deficiency can be obtained by analysing plasma methionine (Met) and cystathionine, the latter by sensitive LC-MS/MS or GC-MS/MS methods. Patients with CBS deficiency have low to low normal cystathionine (reference range typically between 0.05-0.08 and 0.35-0.5 μmol/L) and high to high normal methionine concentrations (reference range typically between 12-15 and 40-45 μmol/L) with a grossly abnormal ratio of these two metabolites. In contrast, genetic and nutritional disorders of Hcy remethylation lead to raised plasma cystathionine and low or low normal methionine concentrations (Orendac et al 2003; Stabler et al 2013; Bartl et al 2014).

#### Maximizing the sensitivity of biochemical testing (statement #5: grade of recommendation C)

*The diagnosis can be masked in patients with mild disease who are taking pyridoxine or pyridoxine-fortified multivitamins and foods prior to biochemical testing*.

The major confounder that may mask the biochemical hallmarks of CBS deficiency is the intake of pyridoxine. Decreases in the tHcy concentration occur after pharmacological doses of pyridoxine in a substantial proportion of CBS deficient patients (Mudd et al 1985; Wilcken and Wilcken 1997; Magner et al 2011). In pyridoxine-responsive patients with some specific mutations (e.g. p.P49L), physiological doses of pyridoxine as low as 2 mg per day in an adult may decrease the tHcy concentrations into the reference range (Stabler et al 2013). Since pyridoxine is contained in many vitamin supplements as well as in fortified foods and drinks, it is important to avoid intake of any pyridoxine supplements for at least 2 weeks before sampling plasma for tHcy measurement, although occasionally a wash-out period of up to 1-2 months may be needed (Orendac et al 2003; Stabler et al 2013).

#### Pre-analytical requirements for biochemical testing (statement #6: grade of recommendation B)

*Plasma should ideally be separated from whole blood within one hour of venepuncture and then can be stored at 4* °*C for up to 1-2 weeks prior to analysis. Although many factors affect the physiology and measurement of tHcy*, *including sample separation and storage*, *most are unlikely to influence the diagnosis of CBS deficiency. Total homocysteine measurement in dried blood spots* (*DBS*) *can be used as a screening test for CBS deficiency if adequate plasma processing is not possible*.

The recommended blood sample handling for measuring plasma tHcy is as follows. Venous blood should be drawn into an anticoagulant specified by the laboratory. This is usually EDTA but some laboratories use heparin or citrate tubes. The sample should be centrifuged within 1 hour if stored at room temperature since red blood cells generate Hcy at a rate of about 1-2 μmol/L/hr in unseparated whole blood (Refsum et al 2004a, b) or within 8 hours if blood with anticoagulants is stored at 4 °C; alternatively, serum may be used (Refsum et al 2004a, b). After centrifugation the tHcy in plasma or serum is stable for at least 4 days at room temperature, for several weeks at 4 °C and several years at -20 °C (Refsum et al 2004a, b). Whilst strict observation of pre-analytical conditions may be important for research studies, differences in plasma tHcy concentrations due to suboptimal pre-analytical procedures or diurnal variation, fed state, pregnancy or posture are relatively minor and unlikely to compromise the diagnosing of CBS deficiency in typical cases (Refsum et al 2004a, b).

An alternative screening approach for determining tHcy is the analysis of DBS obtained by sampling capillary or venous blood on cards used in neonatal screening (McCann et al 2003; Bowron et al 2005; Turgeon et al 2010; Alodaib et al 2012; Bartl et al 2014). This is especially useful in clinical situations when pre-analytical and sample transport conditions cannot be met. Laboratories need to establish a local reference range for tHcy concentrations in DBS, which are 30-40 % lower compared to plasma, due to lower concentration of tHcy in erythrocytes. tHcy levels are stable for a week at room temperature but a 9 % decrease was observed after 28 days (Bowron et al 2005; Bartl et al 2014).

#### Other causes of hyperhomocystinaemia (statement #7: grade of recommendation C)

*Other causes of hyperhomocystinaemia include renal failure*, *nutritional vitamin B*_*12*_ *and folate deficiencies* *and genetic disorders of vitamin B*_*12*_ *absorption or the Hcy remethylation pathway. Decreased or low normal plasma Met*, *elevated plasma cystathionine and*/*or elevated plasma or urinary methylmalonic acid concentrations suggest causes of hyperhomocysteinemia other than CBS deficiency*.

Increased plasma tHcy concentrations are not specific for CBS deficiency as there are many other genetic, nutritional and pharmacological factors as well as several diseases associated with tHcy elevation (Refsum et al 2004a, b). Confirmation of CBS deficiency (as specified below) should be accompanied by excluding other causes of hyperhomocysteinemia; the balance of these two approaches depends on the degree of clinical suspicion of CBS deficiency.

Nutritional causes of hyperhomocysteinemia are common, notably vitamin B_12_ deficiency and, less often, folate deficiency. These should be excluded by measuring serum vitamin B_12_ and/or transcobalamin II, plasma or urine methylmalonic acid and serum folates (Refsum et al 2004a, b). Patients with vitamin B_12_ deficiency can have tHcy up to 450 μmol/L (Stabler 2013). Folate deficiency is particularly likely to cause elevated tHcy concentrations in subjects who are homozygous for the common c.677C > T variant in the *MTHFR* gene. Renal failure is another frequent cause of hyperhomocysteinemia and should be excluded by measuring the serum creatinine concentration. The patient’s history is also important as it may reveal other diseases associated with hyperhomocysteinemia or the administration of drugs such as nitrous oxide, methotrexate and other folate antagonists (Rasmussen and Moller 2000; Refsum et al 2004a, b).

Analysis of additional metabolites can usually distinguish CBS deficiency from genetic and nutritional disorders in the Hcy remethylation pathway. Low normal or decreased plasma Met concentrations and elevated plasma cystathionine (determined by LC-MS/MS or GC-MS) indicate a disturbance in the remethylation pathway; simultaneous elevation of methylmalonic acid in plasma and/or urine suggests more specifically disorders of vitamin B_12_ supply, transport or intracellular metabolism with impaired synthesis of both methylcobalamin and adenosylcobalamin (Stabler 2013; Stabler et al 2013).

#### Confirmatory testing (statement #8: grade of recommendation B-C)

*CBS deficiency should be confirmed by measurement of cystathionine synthase activity in fibroblasts or plasma and*/*or by mutation analysis of the CBS gene. Neither technique can be relied on to demonstrate abnormalities in all cases*.

Confirmation of CBS deficiency cannot be based on a single method as each technique gives normal results in some patients with CBS deficiency. The gold standard for confirming CBS deficiency is generally considered to be the determination of cystathionine production from Hcy and serine in cultured fibroblasts using radioactive or deuterium labelled substrates (Kraus 1987; Smith et al 2012). The fibroblast CBS activity may, however, be normal in mild forms of the disease, despite biochemical and clinical abnormalities and mutations in the CBS gene. In one study, three of 13 CBS deficient patients had normal CBS activity in fibroblasts (Mendes et al 2013). Recently two rapid stable isotope assays have been developed for measuring activity of CBS released from liver and other organs into plasma (Krijt et al 2011; Alcaide et al 2015). Studies on patients with 27 different genotypes showed that sensitivity of the plasma assay was 100 % for detecting pyridoxine non-responsive patients but only 86 % for the pyridoxine responders. Sequencing of the CBS gene is considered the gold standard in molecular diagnostics; however, pathogenic variants may not be detected in one of the parental alleles in up to 7-10 % of CBS deficient patients (Gaustadnes et al 2002; Magner et al 2011). In summary, if one of these techniques (enzyme or DNA analysis) does not confirm a diagnosis of CBS deficiency the other test should be done in a patient with metabolite abnormalities suggestive of this disease.

#### Role of DNA analysis (statement #9: grade of recommendation B)

*Molecular genetic analysis of the CBS gene is helpful for the confirmation of CBS deficiency and for carrier and prenatal testing*.

The reliability of DNA testing depends on the method, technical quality and extent of analysed regions of the CBS gene and on the nature of the underlying mutation (Katsanis and Katsanis 2013). Technical pitfalls include the PCR step that may amplify only one of the parental alleles, failure to detect larger rearrangements and inadequate sequencing of portions of the gene.

Interpreting the results of mutation analysis is straightforward if the genetic variant is already known and segregates with the biochemical and/or clinical phenotype. However, novel variants of unknown significance detected in genomic DNA may be difficult to interpret, especially if they affect RNA splicing and/or its stability. Expression of mutants in heterologous systems may not shed light on pathogenicity of missense variants, as many CBS mutants exhibit normal or even supra-normal catalytic activity and only subtle conformational changes can explain their pathogenicity *in vivo* (Krijt et al 2011; Hnizda et al 2012).

Targeted mutation analysis in individuals at risk for genetic variants segregating in the family is suitable for detecting heterozygotes and for prenatal testing using chorionic villi or amniocytes.

#### Genotype-phenotype correlations (statement #10: grade of recommendation C)

*Over 160 disease causing genetic variants in the CBS gene are known. For compound heterozygotes it is difficult to establish clear genotype*/*phenotype correlations. For a few mutations commonly present in the homozygous state*, *however*, *there are well established genotype*/*phenotype correlations with good concordance between pyridoxine responsiveness and a milder clinical phenotype*.

As of January 2016, more than 900 *CBS* alleles in patients of varied ethnic origin were characterised and 164 different pathogenic genetic variants were observed (www.cbs.lf1.cuni.cz). In some populations specific mutations have been repeatedly detected such as the c.919G > A (p.G307S) in the Irish (Yap 2012), the c.572C > T (p.T191M) in Spanish, Portuguese and South Americans (Cozar et al 2011; Alcaide et al 2015) and the c.1006C > T (p.R336C) in the Qatari population (Gan-Schreier et al 2010), all causing a severe pyridoxine non-responsive form of disease when inherited in the homozygous state. In contrast, the commonest variant, c.833 T > C (p.I278T), has been detected in many European populations and patients from elsewhere who may have European ancestry; when homozygous, this variant leads to a mild pyridoxine-responsive type of CBS deficiency (Skovby et al 2010). Compound heterozygotes carrying the c.833 T > C (p.I278T) variant at one allele are usually at least partially pyridoxine responsive but there are exceptions (www.cbs.lf1.cuni.cz). Genotype/phenotype correlation has been established for additional mutations in homozygous patients, however, such correlations are difficult to infer in individuals who are compound heterozygotes.

#### Prenatal diagnosis (statement #11: grade of recommendation C-D)

*Molecular analysis is the preferred technique for the first-trimester prenatal diagnosis if the mutations in both parents are known. Alternatively*, *enzyme analysis can be performed in cultured amniocytes but not in chorionic villi. Pre-implantation genetic testing is feasible*.

Targeted mutation analysis in native or cultured chorionic villi or in amniocytes can be performed if the mutations are known in the propositus in the family and in both parents. If the mutations in the family at risk are unknown (or known in only one parent) prenatal testing is feasible by determining CBS activity in cultured amniocytes. Native chorionic villi are not suitable for enzymatic diagnosis due to very low activity in controls (Fowler et al 1989) and cultured chorionic villi have also proved unsuitable (Kraus JP, personal communication).

#### Newborn screening (statement #12: grade of recommendation C)

*Newborn screening for CBS deficiency can be performed by detecting elevated Met*, *Met-to-phenylalanine ratio and/or hyperhomocysteinemia in DBS although tHcy has only exceptionally been used as a primary marker. Sensitivity of Met as a primary marker for pyridoxine non-responsive CBS deficiency is limited and inversely related to the chosen cut-off concentrations of Met. For the pyridoxine-responsive form of the disease*, *sensitivity is largely unknown and probably very low. Specificity of Met as a primary marker may be substantially increased by analysing tHcy as a second tier marker and calculating the Met*/*tHcy ratio*.

Long-term experience with newborn screening (NBS) for CBS deficiency has been obtained in a limited number of screening programmes by detecting increased Met concentration in DBS (Chace et al 1996; Mudd 2011). Due to the longer analytical procedure and higher costs, measurement of tHcy as a primary NBS marker has been used so far only in Qatar (Gan-Schreier et al 2010). Molecular genetic testing is feasible and may be an option for high-risk populations with a limited number of prevalent mutations although patients with mutations not contained in the panel will be missed (Gan-Schreier et al 2010). A detailed analysis of newborn screening for CBS deficiency has been published recently (Huemer et al 2015).

While the median Met concentration of 103 μmol/L in DBS of CBS deficient patients are substantially higher than the median of 20 μmol/L in healthy neonates, individual Met values may vary widely (McHugh et al 2011). The sensitivity of Met for detecting newborns with CBS deficiency is not exactly known and 20 %-50 % of pyridoxine non-responsive cases may be missed (Naughten et al 1998; Gan-Schreier et al 2010). Although the pyridoxine responsive form of CBS deficiency represented 44 % of patients in the largest cohort reported so far (Mudd et al 1985), Met most likely fails to detect the majority of pyridoxine responsive patients (Mudd 2011). Indeed, some experts have suggested that screening detects even fewer pyridoxine-responsive cases and that some cases originally thought to respond were incorrectly assigned. Reducing the Met cut-off concentrations increases the detection rate of CBS deficiency and consequently Met cut-off values ranging between 39 and 50 μmol/L have been proposed to increase sensitivity (Turgeon et al 2010; McHugh et al 2011). The sensitivity may also be increased by determining the Met/Phe ratio to adjust for protein intake.

Elevated blood methionine concentrations occur not only in CBS deficiency but also in liver disease, methionine adenosyltransferase I/III deficiency and several other inborn errors of metabolism (Mudd 2011). Low specificity of Met as the first tier analyte can be substantially improved by quantifying tHcy as a second-tier marker (Turgeon et al 2010; Mudd 2011; Alodaib et al 2012). This approach can reduce the false positive rate fivefold (Turgeon et al 2010) and data on tHcy in DBS as a secondary marker showed a good sensitivity with clear distinction between 31 CBS deficient patients (median tHcy 40 μmol/L) and controls (6 μmol/L). In addition, calculating the Met/tHcy ratio may discriminate between CBS and MATI/III deficiency, although more experience with this marker is needed.

#### Family screening (statement #13: grade of recommendation D)

*When an index case is detected*, *screening of at-risk family members should be offered using biochemical testing*.

Since CBS deficiency is an autosomal recessive condition, family members at risk for the disease should be tested by measuring tHcy or, in exceptional cases, by molecular genetic or enzymatic analysis.

### Treatment targets

#### Clinical targets of therapy (statement #14: grade of recommendation D)

*For early diagnosed patients*, *treatment can realistically aim to prevent all the complications of CBS deficiency*, *whilst maintaining normal growth and nutrition* *and allowing the patient normal opportunities for employment and family life. For late-diagnosed patients*, *the aim is to prevent further complications*, *especially thromboembolic disease*.

The clinical outcomes have been studied extensively in Irish, British and Australian patients with CBS deficiency detected by NBS. These studies found that good compliance with dietary treatment prevented ectopia lentis, osteoporosis and thromboembolic events (Wilcken and Turner 1978; Wilcken and Wilcken 1997; Yap and Naughten 1998; Yap et al 2000; Yap et al 2001a, b, c); it also led to normal intelligence. Thus, prevention of all recognised complications is a realistic goal for early diagnosed patients with CBS deficiency. The dietary treatment should not compromise growth and nutrition. This should allow patients to achieve satisfying employment and to have a family if they wish.

Unless diagnosed by NBS or because of a family history, patients will present with one of the complications of homocystinuria. Children are often diagnosed following dislocation of the optic lens, by which time they may have some learning difficulties. Adults are usually diagnosed after a thromboembolic event. For late-diagnosed patients, the main aim is to prevent further complications; behavioural and intellectual improvement has also been reported (Grobe 1980). A major goal is to prevent thromboembolic disease, as this is the commonest late complication (Wilcken and Wilcken 1997; Yap et al 2001a, b, c).

#### Biochemical targets of therapy (statement #15: grade of recommendation C)

*Treatment aims to lower the plasma tHcy concentration to a safe level whilst maintaining normal nutrition*, *including normal concentrations of methionine and other essential AA. In pyridoxine-responsive patients the target for plasma tHcy should be* <*50 μmol*/*L. This may not be achievable for patients that only show a partial response. In pyridoxine-unresponsive patients*, *historical data suggests that good outcomes can be achieved if plasma fHcy levels are maintained below 11 μmol*/*L. This corresponds to a tHcy concentration of about 120 μmol*/*L. We recommend keeping tHcy levels below 100 μmol*/*L but this may need revision when very long term data become available. It is particularly important to remind adolescents and young adults of the dire consequences of poor compliance*.

In general, the aim is to keep the Hcy concentration as close to normal as possible. In patients who are fully-responsive to pyridoxine, standard doses can lead to tHcy levels below 50 μmol/L (and sometimes within the normal range). Some patients who are partially-responsive to pyridoxine may be able to achieve a tHcy level below 50 μmol/L if they are also on a low-Met diet; for others it is not a realistic goal. Excessive Met restriction, with plasma Met concentrations that are sometimes below the normal range, may impair growth and neurodevelopmental progress in children.

The published Irish studies found that pyridoxine-unresponsive patients had a normal IQ and no complications if their lifetime median plasma fHcy concentration was less than 11 μmol/L (Yap and Naughten 1998; Yap et al 2001a, b, c). Some of these patients have subsequently suffered complications, especially thromboembolic events but these followed poor control for many months. There have still been no complications in those Irish patients detected by NBS, now aged up to 43 years, whose plasma fHcy has consistently been less than 11 μmol/L, or who have only had brief rises above this for days or weeks (Crushell E and O’Sullivan S, personal communication).

No long term data relating tHcy concentrations to outcome have been published and it is not possible to predict the tHcy accurately from the fHcy due to the inherent variability in the measurement of fHcy and the wide confidence interval, as discussed above (see Biochemical diagnosis (statement #4: grade of recommendation C)). Using segmented linear regression analysis on the data presented in Fig. 2, we estimate that a median fHcy of 11 μmol/L corresponds to a median tHcy of 120 μmol/L (Fig. 3-inset). Thus, the Irish data suggest that patients may be safe with plasma tHcy levels up to 120 μmol/L.

Within the GDG, there were minor differences of opinion concerning target plasma tHcy concentrations for patients on dietary treatment. Some GDG members thought the aim should be to keep tHcy concentrations below 120 μmol/L because there is evidence to support this; lower target levels are hard to achieve consistently in young adults and may even risk compromising nutrition. Other GDG members favoured stricter targets (e.g. below 70 or 80 μmol/L), as there are fluctuations even in the most compliant of patients and we still do not have very long term outcome data. Following discussion, the GDG thought it was reasonable to recommend keeping the plasma tHcy concentration below 100 μmol/L; stricter control may have benefits but this is unproven.

As discussed in Pre-analytical requirements for biochemical testing (statement #6: grade of recommendation B), tHcy concentrations are lower in DBS. If these are used for monitoring, the target level should be adjusted appropriately (below about 60-70 μmol/L, depending on the method used).

Particular efforts should be made to keep tHcy levels in the target range in adolescents and young adults and patients must be made aware of the dire consequences of poor compliance. Thromboembolism is the main concern; high tHcy levels at this age may also lead to lens subluxation and possibly psychiatric issues.

### Pyridoxine-responsive homocystinuria

#### Assessment of pyridoxine-responsiveness (statement #16: grade of recommendation C-D)

*To assess pyridoxine responsiveness after infancy*, *we recommend giving 10 mg*/*kg*/*day pyridoxine up to a maximum of 500 mg*/*day for 6 weeks*; *the plasma tHcy concentration should be measured at least twice before treatment and twice on treatment. The test should not be done if the patient is catabolic. The protein intake should be normal*, *folate supplements should be given and vitamin B*_*12*_ *deficiency should be corrected prior to testing. Patients who achieve plasma tHcy levels below 50 μmol*/*l on pyridoxine are clearly responsive and do not need any other treatment. If the tHcy falls* >*20* % *but remains above 50 μmol*/*L*, *additional treatment should be considered* (*i.e. diet and*/*or betaine*). *If tHcy falls by* <*20* % *on pyridoxine*, *the patient is likely to be unresponsive*.

*Patients detected by newborn screening rarely respond to pyridoxine and*, *in this group*, *we recommend using a relatively high dose* (*100 mg*/*day*) *for a shorter period* (*2 weeks*).

Pyridoxine responsiveness is often defined according to the plasma concentration of homocysteine (or related measurements) on treatment. Criteria have included tHcy <50 μmol/L (Kluijtmans et al 1999), fHcy not detectable (i.e. approximately tHcy <55 μmol/L, as discussed above) (Yap et al 2001a, b, c), fHcy <10 μmol/L (Walter et al 1998) or total free homocysteine (i.e. twice homocystine concentration plus homocysteine-cysteine disulfide concentration) <20 μmol/L (Wilcken and Wilcken 1997). Many patients, however, show a partial response to pyridoxine (Brenton and Cusworth 1971) and it should be continued in these patients. Additional treatment is needed if there is a partial response and target levels are not reached.

**Fig. 4:**
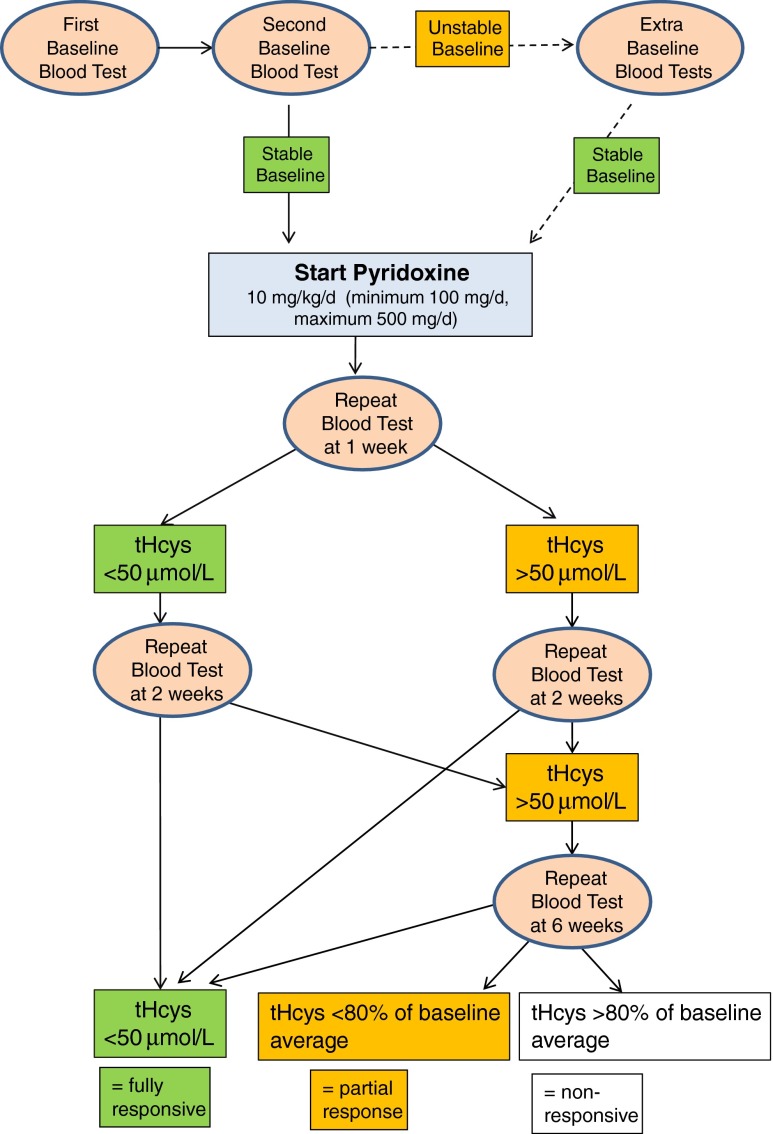
Proposal for assessing pyridoxine responsiveness after infancy. The baseline must be stable and should be the average of at least two separate measurements

Various protocols have been used to test pyridoxine responsiveness (Mudd et al 1995; Wilcken and Wilcken 1997; Walter et al 1998; Yap and Naughten 1998; Kluijtmans et al 1999; Yap et al 2001a, b, c). Our recommendation (Fig. 4) takes account of physiological variation in tHcy levels and the limited precision of measurements. Folate and vitamin B_12_ deficiencies can impair the response to pyridoxine and some patients take several weeks to achieve their full response (Walter et al 1998; Yap et al 2001a, b, c).

Patients detected by newborn screening seldom respond to pyridoxine (Morrow and Barness 1972; Wilcken and Turner 1973; Kluijtmans et al 1999). Unpublished data from the E-HOD registry, however, show that sensitive screening strategies may detect more patients with the pyridoxine-responsive form of homocystinuria than reported previously (Huemer and Kozich, unpublished). To avoid delaying effective treatment, we recommend giving neonates a relatively high pyridoxine dose (e.g. 100 mg/day) for at least 2 weeks, with measurement of the plasma tHcy at the end of the first and second weeks. If a patient shows an equivocal fall in Hcy, the trial should be continued for longer, to confirm that the fall is not just due to anabolism. If cystathionine is measured, a rise in this metabolite provides extra evidence for a genuine response to pyridoxine.

#### Adverse effects of pyridoxine (statement #17: grade of recommendation D)

*Peripheral neuropathy is the most important adverse effect of pyridoxine. It has been reported in a number of patients treated with long-term high doses of pyridoxine* >*900 mg*/*day*.

There is a high risk of peripheral neuropathy following long-term treatment with pyridoxine doses above 900 mg/day (Schaumburg et al 1983; Ludolph et al 1991, 1993), but it has not been found in patients treated with less than 500 mg/day (Cohen and Bendich 1986; Mpofu et al 1991; Yap et al 2001a, b, c). Reports of peripheral neuropathy with doses below 500 mg/day are unreliable (Parry and Bredesen 1985). Withdrawal of pyridoxine has led to improvement of the neuropathy in some patients (Schaumburg et al 1983).

Periods of apnoea and unresponsiveness have been reported in a few neonates following large oral doses of pyridoxine (500 mg/d) (Mudd et al 1995), as well as after intravenous doses for pyridoxine dependent epilepsy. Rhabdomyolysis has also been reported (Shoji et al 1998).

#### Recommended pyridoxine doses (statement #18: grade of recommendation D)

*For long-term treatment*, *the pyridoxine dose should be the lowest that achieves the biochemical targets* (*plasma total homocysteine* <*50 μmol*/*L*). *We recommend using doses up to 10 mg*/*kg*/*day and avoiding doses above 500 mg*/*day*.

Pyridoxine doses of 10-40 mg can achieve biochemical targets in some patients with the p.P49L and p.I278T mutations (Stabler et al 2013). Pyridoxine doses of 200 mg/day or less can achieve the biochemical targets in many other patients. Partially pyridoxine-responsive patients need higher doses and additional treatment (betaine and/or diet). The risk of peripheral neuropathy appears to be low in adults with pyridoxine doses below 500 mg/day (see Adverse effects of pyridoxine (statement #17: grade of recommendation D)). In children, the safe dose is likely to depend on body weight; there are few data but we suggest using doses up to 10 mg/kg/day, with a maximum of 500 mg/day.

#### Pyridoxine in non-responsive patients (statement #19: grade of recommendation D)

*There is no evidence that long-term pyridoxine is beneficial if there is no biochemical response in a properly conducted test*.

There is no evidence that pyridoxine has beneficial effects independent of lowering Hcy concentrations. Though there can be a delay in seeing the full response, some response (i.e. >20 % decrease) should be seen within two weeks in patients who are not deficient in vitamin B_12_ or folate.

### Role of vitamin B_12_ and folate supplementation (statement #20: grade of recommendation D)

*All patients should receive adequate folate supplementation. Vitamin B*_*12*_ *should be monitored and supplemented if deficient*.

There are several reports of vitamin B_12_ and folate deficiencies in patients with CBS deficiency (Smolin et al 1981; Ishida et al 2001). This may be due to increased flux through the remethylation pathway and use of the cofactors, or inadequate intake of the vitamins in patients on restricted diets. We recommend giving all patients low-dose folate supplements and monitoring their vitamin B_12_ levels. High dose folate therapy may lead to an additional benefit through enhancing the remethylation pathway but may have side effects (Wang et al 2014). Though it is clear that folate deficiency must be avoided, there is little evidence concerning the optimal dose of folate supplementation. In patients on dietary treatment, folate and vitamin B_12_ supplements are generally included in the Met-free L-AA supplement, and it is not clear whether additional supplements are routinely required.

### Dietary management

#### Approaches to dietary treatment (statement #21: grade of recommendation C-D)

*Dietary treatment should be considered for all patients with CBS deficiency unless target Hcy levels are achieved entirely by pyridoxine supplementation. Diet may be used either as a sole treatment or adjunctive therapy along with pyridoxine and*/*or betaine. Most pyridoxine-unresponsive patients require a diet that is very low in natural protein*, *with supplements of a Met-free L-AA mixture. Lifelong treatment is required*.

Dietary management of CBS deficiency can be highly successful. It should be considered for all pyridoxine unresponsive patients and as additional treatment in individuals who are partially pyridoxine responsive (Komrower et al 1966; Perry et al 1966, 1968; Mudd et al 1985; Pullon 1988; Walter et al 1998; Yap and Naughten 1998; Lutteri et al 1999; Kabra 2002; Keating et al 2011; Schiff and Blom 2012; Adam et al 2013; de Lonlay et al 2002).

Restricting intake of the essential AA, Met, reduces the precursor load on the transsulfuration pathway, thereby reducing Hcy production. In most pyridoxine-unresponsive patients, the biochemical targets can only be achieved by a diet that is very low in natural protein, with supplements of a Met-free L-AA mixture. The approach is analogous to the management of phenylketonuria (PKU) for which there is a greater body of published evidence.

There are very few reported complications with well managed dietary treatment (Perry et al 1968), however the diet is complex and difficult so poor adherence is common (Lawson-Yuen and Levy 2006; Schiff and Blom 2012). Problems can be reduced by starting dietary treatment in individuals as young as possible and utilising the skills of an experienced metabolic dietitian (Yap and Naughten 1998; Kabra 2002; Schiff and Blom 2012). Treatment for CBS deficiency must be continued throughout life, as loss of biochemical control in later life is associated with serious complications (Walter et al 1998). Compliance with treatment often deteriorates, particularly in adolescence, as in other disorders (Walter et al 1998). Initiating dietary restrictions in late diagnosed individuals is more challenging than in neonates but it can reduce the risk of further complications and lead to improvement, for example in seizures and behaviour (Holliday et al 1976; Walter et al 1998; Garland et al 1999; Kabra 2002).

Additional treatment with betaine can help patients who find it difficult to adhere to dietary restrictions and to attain good metabolic control (see Betaine treatment). Betaine lowers Hcy levels, potentially allowing an increase in Met intake (Walter et al 1998). Met restriction in individuals treated with betaine can also prevent excessively raised Met levels and the possible risks associated with these—see Side effects of betaine (statement #28: grade of recommendation C-D) (Pullon 1988; Garland et al 1999; Lawson-Yuen and Levy 2006). A recent European survey of pyridoxine unresponsive patients found that a combination of dietary restriction and betaine was the commonest treatment (Adam et al 2013).

#### Methionine restriction (statement #22: grade of recommendation D)

*The level of Met or natural protein restriction required varies and is determined for each patient according to their plasma tHcy and Met concentrations*.

The target plasma tHcy concentrations are discussed in Biochemical targets of therapy (statement #15: Ggrade of recommendation C). The amount of Met that a patient can take whilst achieving these levels depends on various factors, including the degree of residual CBS enzyme activity, the responsiveness to pyridoxine, the use of betaine and the patient’s age and growth rate. The allowance of Met (or natural protein) should be determined for each patient individually by an experienced metabolic dietitian, based on their plasma Met and tHcy levels.

Met is an essential AA and an adequate supply should be ensured, particularly in growing children. There are, however, no data for the Met requirement in patients with CBS deficiency. Recommendations by the WHO cannot be used as they are expressed for Met and cysteine combined (WHO et al 2007). Close monitoring of plasma Met levels and growth are needed in patients with severe CBS deficiency, who require a low Met intake in order to achieve good biochemical control.

For infants diagnosed through NBS in the UK, the supply of Met/natural protein (from breast milk or formula) is stopped (after the pyridoxine test) and a Met-free complete infant formula is given for 2-4 days to reduce Hcy levels. Met in the form of breast milk or infant formula is then introduced, divided into several feeds, in conjunction with the Met-free formula (Dixon et al 2015). UK guidelines recommend a starting allowance of 90-120 mg Met/day (or 30 mg/kg/day if weight <3 kg). The Met allowance is then titrated against the patient’s plasma tHcy levels. A potential management pathway is shown at http://www.bimdg.org.uk/store/enbs//HCU_Dietetic_Management_Pathway_V1_April_2015_215380_12052015.pdf.

Breast feeding has proven benefits for infants but there are few published reports of its use in inherited metabolic diseases other than PKU. An international survey reported five infants with CBS deficiency who had received breast-feeds in combination with a Met-free AA infant formula (MacDonald et al 2006a, b). The principles are the same as for breast-feeding in PKU (Francis and Smith 1981). Measured volumes of a Met-free infant formula are given prior to breast-feeds, thereby limiting the amount of breast milk (and Met) taken.

Weaning should begin at the usual time and progress through stages as normal. Naturally low protein foods or manufactured low protein foods are introduced first. Gradually the Met allowance of breast milk or standard formula is replaced with protein/Met containing foods. Age appropriate concentrated L-AA supplements are introduced at appropriate times to ensure full protein requirements are met.

For older children and adults, the diet is either based on Met-restriction or natural protein-restriction. The Met-restricted diet is calculated by estimating the daily Met intake, whereas the protein-restricted diet is based on the intake of natural protein and the assumption that 1 g of protein provides a specific quantity of Met. For practical reasons, a combination of the two is often used (Adam et al 2013). In theory, the Met-restricted diet is preferable because the Met content of different foods varies relative to their protein content. There are, however, limited data for the Met content of foods (Adam et al 2013).

The introduction of dietary restriction is extremely difficult in late diagnosed patients and is best done gradually. If a Met-free L-AA supplement is likely to be required, this should be started first to ensure nutritional adequacy whilst reducing the Met/protein intake.

#### Role of L-AA mixtures (statement #23: grade of recommendation D)

*The majority of patients on dietary treatment require a low Met diet with a cystine-enriched, Met-free L-AA supplement. Some partially pyridoxine-responsive patients benefit from milder protein restriction without Met-free L-AA supplements*.

In CBS deficient individuals, the natural protein/Met allowance from food may be too low to support a normal growth rate in infants and children and to meet protein requirements in adults. In these instances a cystine-containing, Met-free L-AA formula/supplement should be used. These are also often supplemented with fat, carbohydrate and micronutrients and are available in a variety of forms with age-specific micronutrient provision (Walter et al 1998). In a recent European dietetic survey, all children <16 years were prescribed an age-appropriate L-AA supplement (Adam et al 2013).

There are currently no specific recommendations for the Met-free L-AA or total protein intake in CBS deficiency (Adam et al 2013). The WHO/FAO/UNU (2007) recommendations can be used to guide the total protein requirements (WHO et al 2007) but PKU research suggests that higher protein intakes than the WHO recommendations are required to compensate for the sub-optimal bio-availability of L-AAs in current preparations (MacDonald et al 2006a, b). It is recommended that the L-AA supplement is split into three to four doses throughout the day to maximise nitrogen retention and achieve appropriate growth (Acosta 2010).

It is important to avoid both inadequate and excessive intake of the L-AA supplement. Inadequate intake may be due to being insufficiently prescribed or poor adherence and can result in poor biochemical control, poor growth and malnutrition (Walter et al 1998). Adherence to Met-free L-AA supplements can be poor due to their taste.

Patients who are partially pyridoxine-responsive may achieve target tHcy levels with a relatively mild protein restriction and may not require Met-free L-AAs supplements. Late-diagnosed pyridoxine-unresponsive patients are also sometimes managed with a relatively mild protein restriction and betaine, if they cannot adhere to a stricter diet. These patients still require close monitoring of their nutritional status and dietary intake.

#### Role of cysteine. (statement #24: grade of recommendation D)

*In patients with severe deficiency*, *additional cystine supplementation may be necessary but there is no evidence to guide the level required*.

Cysteine is normally formed from methionine via the transsulfuration pathway. Cysteine is, therefore, a ‘conditionally essential’ AA in CBS deficiency and low concentrations may contribute to the pathogenesis. Cysteine, like Hcy, contains a thiol group and is present in different reduced and oxidised forms. Since the binding capacity of plasma proteins for thiols is limited, grossly elevated Hcy concentrations lead to a decrease in plasma total cysteine, as often seen in poorly controlled patients. The emphasis should, however, be on improving Hcy control as total cysteine levels increase when Hcy levels fall (see also Fig. 2). Results should be interpreted in liaison with the monitoring laboratory, as most laboratories measure free (non-protein-bound) cystine concentrations, which may be falsely low without prompt deproteinisation of the blood samples (Hargreaves et al 2002). Total cysteine measurement avoids these preanalytical complications but it is not widely available.

Case reports suggest that cysteine deficiency can cause poor weight gain and growth even in the presence of adequate energy intake (Perry et al 1966). Cystine is added to most Met-free L-AA supplements but the quantities are sometimes no greater than in supplements for other AA disorders, such as PKU. It is not clear if this is sufficient. Cystine supplementation can be difficult to administer due to its poor solubility and unpleasant taste.

Further research is needed addressing the accurate cysteine levels in treated patients and whether additional L-cystine supplementation improves the outcomes of patients with good Hcy control.

#### Role of energy and micronutrient supplements (statement #25: grade of recommendation D)

*The diet should be nutritionally complete whilst being varied and palatable*, *to optimise adherence*.

It is important that energy requirements are met to ensure optimal dietary protein utilisation as protein synthesis and catabolism are energy dependent. National recommendations for energy intake should be followed because the specific energy requirement in CBS deficiency is unknown. Manufactured low protein foods are available and most European metabolic centres use them to ensure adequate energy intake in CBS deficiency (Adam et al 2013). Studies in other metabolic disorders suggest that low protein foods improve compliance with the diet and palatability, although there is no specific evidence for this in CBS deficiency.

There are no established micronutrient requirements for CBS deficiency and normal population reference values should be used as a guide. If micronutrients are not included within the L-AA supplement, additional supplements may be required. The intake should be assessed regularly, particularly for calcium, and micronutrient status should be monitored.

Many L-AA supplements now contain long chain polyunsaturated fatty acids, as strict low protein/Met diets are usually deficient in essential fatty acids. Separate supplements could be considered if they are not included in the L-AA supplement. There are no reports of deficiency in CBS deficiency.

### Betaine treatment

#### Role of betaine (statement #26: grade of recommendation C-D)

*Betaine should be considered as adjunctive treatment in patients who cannot achieve target levels of Hcy by other means*.

Betaine (N,N,N-trimethylglycine) is formed in the body from choline and small amounts are present in the normal diet (Zeisel et al 2003). It lowers Hcy concentrations in CBS deficiency by donating a methyl group and converting Hcy to Met (Komrower et al 1979; Smolin et al 1981; Wilcken et al 1983, 1985; Singh et al 2004). Betaine may also act as a chemical chaperone and correct partial mis-folding of *CBS* mutants (Bourot et al 2000; Diamant et al 2001, 2003; Kopecka et al 2011). Betaine can increase cysteine levels (Benevenga 1984; Wilcken et al 1985) but this is probably secondary to decreased protein bound Hcy.

Betaine treatment alone seldom achieves target Hcy levels in patients with pyridoxine-unresponsive CBS deficiency. Studies of CBS-deficient mice gave similar results (Gupta et al 2016). This may be because betaine treatment raises the Met concentration. Individuals with plasma Met concentrations greater than 80 μmol/L respond less well to betaine (Sakamoto and Sakura 2003), though in practice some response is usually seen. For these reasons, betaine is best used as adjunctive treatment in patients who are partially responsive to pyridoxine or who are on dietary treatment but cannot achieve good control.

#### Recommended betaine doses (statement #27: grade of recommendation C-D)

*Patients*’ *responses to betaine are variable and optimal doses have to be individualised. For children*, *the initial betaine dose is 50 mg*/*kg twice daily. For adults*, *the starting dose is 3 grams twice a day. The dose and frequency are adjusted according to response. There is unlikely to be any benefit in exceeding a dose of 150-200 mg/kg/day*.

The published doses of betaine vary and very few studies are consistent. Betaine has a half-life of 14 hours so twice daily dosing is adequate (Schwahn et al 2003).

In children, the initial dose is 100 mg/kg/day, divided into twice daily doses, and then adjusted according to response (typically increased weekly by 50 mg/kg increments). Studies based on pharmacokinetic and pharmacodynamic modelling after single doses of 50-100 mg/kg betaine suggest there is unlikely to be any additional benefit from using doses higher than 150-200 mg/kg/day (Matthews et al 2002; Schwahn et al 2003).

The maximum licensed dose is 3 grams twice daily and this is the usual dose in adults but higher doses have sometimes been used with anecdotal evidence of biochemical benefit.

#### Side effects of betaine (statement #28: grade of recommendation C-D)

*Generally betaine is well tolerated and safe. Higher doses have been associated with a fishy odor. Cerebral edema is a very rare side effect*.

Betaine is generally safe but some people dislike the taste and compliance may be poor (Walter et al 1998). It can result in a fishy odor (Manning et al 2012). This is probably due to inadequate activity of flavin containing monooxygenase 3 and may respond to riboflavin (Manning et al 2012).

Acute cerebral edema has been reported in two CBS deficient patients treated with betaine. The plasma Met concentration was above 2000 μmol/L in one patient (Yaghmai et al 2002) and 1190 μmol/L in the other patient (Devlin et al 2004). In both patients, problems resolved after withdrawing betaine and lowering the plasma Met concentration. Two other patients treated with betaine have developed similar white matter abnormalities without evidence of raised intracranial pressure; their plasma Met concentrations were 904 and 1282 μmol/L. One patient made a full recovery after the plasma Met decreased (Sasai et al 2015); neurological deficits persisted in the other patient, who was encephalopathic for more than 2 months before the plasma Met was lowered (Vatanavicharn et al 2008). A number of other CBS deficient patients on betaine treatment have had Met levels above 1000 μmol/L and have not experienced cerebral edema. Cerebral edema has also been seen in a few non-CBS-deficient patients with high levels of Met. Further research is required but the current recommendation is to avoid Met levels above 1000 μmol/L in patients treated with betaine.

### Monitoring (statement #29: grade of recommendation D)

*Monitoring of plasma tHcy*, *AA*, *folate and vitamin B*_*12*_ *is recommended in all patients. The frequency depends on the severity of CBS deficiency*, *treatment*, *age and clinical condition of the patient. These factors also determine the need for additional monitoring*; *for example*, *patients on dietary treatment require regular nutritional assessment*.

Total homocysteine, plasma AAs (including Met), vitamin B_12_ and folate should be monitored regularly in all patients with CBS deficiency. There is, however, little evidence concerning the optimal frequency of monitoring. This will vary depending on the severity of the disorder (e.g. pyridoxine-responsiveness), the patient’s treatment, compliance, age and previous complications (e.g. thrombosis). In adult patients who are fully pyridoxine-responsive it may be adequate to monitor tHcy levels every six months. In contrast, in children on dietary treatment for pyridoxine-unresponsive CBS deficiency, tHcy and Met will need to be monitored much more frequently.

The method of analysis may also influence the frequency of analysis. If tHcy is monitored in DBS sent in from home, it is reasonable to request samples every week during infancy (as in PKU) but this technique is not yet widely available. In most centres, patients will need to attend a hospital for tHcy monitoring samples to be taken from liquid blood and samples will be taken less often.

The serum vitamin B_12_ and folate levels should be measured annually; if the vitamin B_12_ is low, an intramuscular supplement is generally given and levels repeated every 3-6 months thereafter.

Patients on dietary treatment require regular nutritional assessment and additional tests, depending on the patient’s age and clinical condition. Some suggestions are listed in Table 3. One should consider annual monitoring of the blood count, renal profile, liver profile, copper, zinc and selenium, vitamin D and essential fatty acids as well as plasma AAs. There is no specific evidence relating to CBS deficiency but there are reports of micronutrient deficiency in patients on similar dietary treatment for PKU. Tests should be done more frequently if there is poor adherence to diet, inadequate medical food consumption, poor growth or clinical evidence of malnutrition. More extensive monitoring can be done if clinically indicated. Supplements should be given if nutritional deficiencies are identified.

**Table 3:** Monitoring recommendations

Area	Tests	Frequency
Anthropometry	Height & weight	Every clinic visit
Dietary	Dietary intake analysis	Every clinic visit if on dietary treatment
Biochemical–metabolic control	tHcy, Met	See text
Nutritional	Vitamin B_12_, folate	At least annually
	Blood count, albumin, plasma AA, ferritin, zinc, 25-hydroxyvitamin D	At least annually if on dietary treatment
	Selenium, essential fatty acids	If concerns about intake
Neurodevelopmental/neurological	Clinical examination	Annually
	MRI/EEG	Only if new CNS symptoms
Ophthalmological	Eye examination	At least annually
Neuropsychological ffunction	IQ	At least every 5 years during childhood
Psychological	Clinical psychology or psychiatric assessment	As required
Bone density	DEXA	Every 3-5 years from adolescence—unless clinically indicated earlier
Cardiovascular	Lipid profile, cardiovascular risk factor review	Once in childhood, annually in adulthood

Bone density scans (DEXA) should be done every 3-5 years from adolescence with additional scans in individuals who have frequent fractures and/or low vitamin D levels. Neuroimaging (MRI) is only indicated in individuals who have abnormal neurological signs.

### Complications of CBS deficiency

The major complications of CBS deficiency can be categorised into four groups: ophthalmological, skeletal, neurological and cardiovascular/haematological. Rarer complications include pancreatitis and spontaneous pneumothorax (Franchis et al 1998). The complications can be prevented by treatment; once they have occurred, they are often irreversible but interventions can minimise further problems.

#### Ophthalmological complications and interventions (statement #30: grade of recommendation C)

*Ophthalmological complications in CBS deficiency are common and include myopia*, *ectopia lentis and associated complications* (*glaucoma*, *retinal detachment*). *The true frequency is unknown. Ectopia lentis may be the first and only sign of CBS deficiency. Early diagnosis and lifelong treatment with good biochemical control can prevent this complication. Loss of biochemical control can be associated with progression of ocular complications*, *even in adults*, *and early signs include the development of myopia and iridodonesis. Restoration of biochemical control can halt progression of these complications. Regular assessment by an ophthalmologist is recommended*.

Ectopia lentis is the most consistent clinical finding in CBS deficiency and many cases have been diagnosed because of this (Mudd et al 1985). Lens dislocation is extremely rare in the first 2 years of life but, without treatment, 85 % of pyridoxine non-responders have dislocated lenses by 12 years of age. Lens dislocation also occurs in many pyridoxine-responsive patients, including adults.

Dislocation of the lens results from disruption of the zonulae fibres, which are thickened and abnormal in CBS deficiency. Dislocation is usually bilateral and often occurs inferiorly or nasally, or both (Ramsey et al 1972; Ramsey and Dickson 1975; Yap 2000). Iridodonesis is the quivering of the iris caused by moving the eyeball and is a sign of the loosening of the lens.

Disruption of the zonulae fibres also leads to increased curvature of the lens and thereby to lenticular myopia and astigmatism. High myopia >5D is extremely rare in children under 5 years and probably affects under 2 % of adults (Mohindra and Held 1981). Lenticular subluxation due to homocystinuria should be suspected in these patients if there is no evidence of axial myopia on fundoscopy and corneal curvature is normal. Rapid progression of myopia also suggests a lenticular cause, as does high non-corneal astigmatism with subnormal visual activity (Cruysberg et al 1996).

In addition to myopic astigmatism, lens dislocation can lead to retinal detachment, strabismus and cataracts. Anterior dislocation of the lens can cause acute pupillary block glaucoma (Mudd et al 1995). Complete lens dislocation is associated with increased ocular axial length, which may be an attempt to compensate for the blurred vision (Mulvihill et al 2004).

#### Skeletal complications and interventions (statement #31: grade of recommendation C-D)

*Skeletal complications in CBS deficiency are common and include long bone overgrowth and premature osteoporosis. The true frequency is unknown. Early diagnosis and lifelong treatment with good biochemical control can prevent this complication. Loss of biochemical control can be associated with progression of skeletal complications and early signs include exaggerated growth velocity in children. Regular DEXA scans are recommended from adolescence unless clinically indicated earlier. Osteoporosis should be managed by an appropriate specialist*.

Skeletal changes are common but they are often absent in pyridoxine-responsive individuals. There are no abnormalities at birth (Sorsby et al 1960; Smith 1967; Mudd et al 1985) and they are rare in infants or very young children. Genu valgum and pes cavus are usually the first signs. As puberty nears, dolichostenomelia becomes evident and the limbs grow out of proportion to the trunk. Anterior chest wall deformities, such as pectus excavatum or carinatum, and kyphosis or scoliosis may be present (Brenton et al 1972; Carson 1982). The facial appearance may be altered by prominence and protrusion of the upper teeth due to overcrowding and the palate is almost always high arched (Mudd et al 1995; Brenton et al 1972). These skeletal manifestations give older patients an appearance similar to that of Marfan syndrome (Smith 1967; Morreels et al 1968; Beals 1969; Brenton et al 1972; Schedewie et al 1973; Boers et al 1983, 1984; Mudd et al 1985) but CBS deficiency can be distinguished by the presence of osteoporosis (Carson 1982).

Osteoporosis, especially of the spine, is the most consistent skeletal abnormality. In the largest review, 70 % of untreated pyridoxine-unresponsive patients developed spinal osteoporosis by 16 years of age (Mudd et al 1985). This may, however, be an overestimate as osteoporosis was not defined according to current criteria (ISCD, 2013, http://www.iscd.org/official-positions/2013-iscd-official-positions-pediatric). Spinal osteoporosis also occurs in many pyridoxine responsive cases, usually at a later age (Mudd et al 1985). Early diagnosis and effective treatment reduce the risk of osteoporosis (Yap and Naughten 1998; Lim and Lee 2013).

Osteoporosis may lead to vertebral collapse (Schimke et al 1965; Brenton 1977) and scoliosis, though the latter has also been reported in the absence of osteoporosis. DEXA scanning should routinely be performed every 3-5 years from adolescence unless there is a clinical indication to do this earlier (such as recurrent fractures). Due to the sensitivity of the test, there is little point in repeating this more frequently than every 2 years. In patients with reduced bone mineral density, it is particularly important to check vitamin D status and dietary calcium intake and to encourage weight-bearing exercise.

#### Cardiovascular complications and interventions (statement #32: grade of recommendation C-D)

*Venous thromboembolic complications are common in untreated or poorly controlled CBS deficiency. In untreated pyridoxine unresponsive patients*, *the risk may be as high as 50* % *by 30 years of age. The frequency in pyridoxine responders is unknown. It may be the first and only sign of CBS deficiency. Early diagnosis and lifelong treatment with good biochemical control can prevent this complication. Prevention of dehydration is important. Patients who are poorly controlled or have had a vascular event may warrant additional treatment with anti-platelet drugs or anticoagulants*, *according to local guidelines*.

Vascular complications are common in untreated or poorly controlled CBS deficiency and include venous thrombosis and arterial thrombosis. Venous thrombosis is the more common. In the largest published series of untreated patients, 50 % of the vascular complications were deep venous thrombosis (a quarter of which were associated with pulmonary embolism); stroke (including cerebral venous sinus thrombosis) comprised 32 % of the vascular complications, peripheral arterial disease 11 % and myocardial infarction only 4 % (Mudd et al 1985).

It has been suggested that the risk of thrombosis in homocystinuria depends largely on whether patients also have the factor V Leiden mutation (Mandel et al 1996) but this was not supported by subsequent studies (Kluijtmans 1998, Yap 1999, Gaustadnes 2002). The value of testing for such thrombophilic mutations is, therefore, unknown and possibly low.

In patients with CBS deficiency, lowering the plasma tHcy is the single most important factor in reducing the risk of thromboembolic disease (Wilcken and Wilcken 1997; Yap 2003). Additional preventive treatment should be used if there are other risk factors for thromboembolism, such as immobility due to surgery or travel (Special management issues). Dehydration and infection increase the risk of venous thrombosis, particularly in children, by increasing blood viscosity and all the reports of cerebral venous sinus thrombosis in neonates with CBS deficiency have occurred in the context of severe infections and dehydration (Cardo et al 1999; Karaca et al 2014). It is, therefore, important to ensure patients with CBS deficiency are well hydrated at all times and especially when unwell and during anaesthesia and surgery (see Management of surgery and anaesthesia (statement #38: grade of recommendation C-D)).

There is some evidence that there may be increased platelet activation in patients with CBS deficiency, even on treatment (DiMinno et al 1992). Treatment with antiplatelet drugs such as aspirin, dipyridamole or clopidogrel may, therefore, be indicated in patients who are poorly controlled or have already had a vascular event. Coumarin anticoagulants may be appropriate for patients who have had a venous thrombosis.

Acute stroke can occur in CBS deficiency patients due to arterial thrombosis/embolism and carotid artery disease/dissection but it is usually due to cerebral venous sinus thrombosis (Mudd 1985; Hou and Wang 1994; Kelly et al 2003; Weiss et al 2006; Granel et al 2009). It is important to lower the plasma Hcy level, particularly if the stroke is the presenting feature (Wilcken and Wilcken 1997; Yap 2003). Anticoagulation carries the risk of bleeding into an oedematous brain, especially if there is hypertension, and needs to be decided on an individual basis. In children, particular care should be taken to ensure adequate hydration and to treat any underlying infection (Cardo et al 1999).

#### Neurological and psychological complications and interventions (statement #33: grade of recommendation D)

*Neurological complications include seizures and movement disorders. Treatment can be difficult and should involve a neurologist. Cerebral white matter abnormalities have been reported in a few patients treated with betaine but there is no evidence to support routine MRI or EEG surveillance*, *in the absence of symptoms. Psychological and psychiatric complications*, *including psychosis and depression*, *are common in patients with CBS deficiency*.

Neurological complications include seizures and movement disorders in addition to strokes. Seizures occur in 20 % of untreated pyridoxine-unresponsive patients by 12 years of age. EEG abnormalities may be more common, reflecting underlying brain disease. There is no evidence to support routine EEG monitoring in the absence of symptoms (del Giudice et al 1983; Mudd et al 1985; de Franchis et al 1998; Buoni et al 2003). There are several case reports of movement disorders which are not due to basal ganglia infarction, including polymyoclonus, dystonia and parkinsonism. Some of these patients have responded to L-DOPA therapy but others have not and this drug should be used with caution as it can raise Hcy levels (Kempster et al 1988; Awaad et al 1995; Keskin and Yurdakul 1996; Burlina et al 2002; Ekinci et al 2004; Sinclair et al 2006).

Cerebral white matter abnormalities have been reported in a few patients treated with betaine, associated with high plasma Met concentrations (see Side effects of betaine (statement #28: grade of recommendation C-D)). Routine MRI surveillance is not, however, recommended in patients with CBS deficiency unless they have neurological symptoms (Keskin and Yalcin 1994; Ekinci et al 2004; Vatanavicharn et al 2008; Brenton et al 2012).

Many psychological and psychiatric conditions are over-represented in patients with CBS deficiency. These include psychosis, obsessive-compulsive disorder, depression and behaviour/personality disorders (such as aggression and drug or alcohol abuse) (Grobe 1980; Bracken and Coll 1985; Abbott et al 1987; de Franchis et al 1998; Hidalgo et al 2013).

### Fertility, contraception and pregnancy in CBS deficiency

#### Fertility (statement #34: grade of recommendation C-D)

*There is no compelling evidence that CBS deficiency affects fertility. There is also no convincing evidence that CBS deficient patients have an increased risk of fetal loss or fetal malformations*.

The only published studies looking at fertility in CBS deficiency have been in mice. These showed changes in the estrus cycle, possible oocyte differences, increased fetal loss and lactation disturbances (Guzman et al 2006; Nuno-Ayala et al 2010). Mouse studies also indicate that CBS is important (via its role in H_2_S production) in the maintenance of labour (You et al 2011).

Increased malformations have not been reported in the children of women with CBS deficiency (Levy et al 2002). It is more difficult to analyse fetal loss because it is not always reported and early losses are not always recognised. Although data from several studies link miscarriage to increases in Hcy (Owen et al 1997), studies in treated patients with CBS deficiency have not shown an increased risk of miscarriage compared to the general population (Mudd et al 1985).

Males are fertile and in one study 21 males sired 34 fetuses of which 33 were healthy and one spontaneously aborted (Mudd et al 1985).

#### Contraception (statement #35: grade of recommendation D)

*Estrogen containing contraceptives should be avoided due to the increased risk of thrombosis*.

This recommendation was originally made when contraceptives had a relatively high estrogen content (McCully 1975; Grobe 1978). It is unknown whether lower dose estrogen preparations are safe, for example in well-controlled pyridoxine-responsive CBS deficiency.

#### Pregnancy, delivery and peripartum management (statement #36: grade of recommendation C-D)

*Pregnancy*, *delivery and the post-partum period pose an additional risk of thrombosis in CBS deficient women. Anticoagulant therapy in the form of low molecular weight heparin is recommended during the third trimester* (*and maybe throughout pregnancy*) *and for at least 6 weeks postpartum. Met requirements often increase through pregnancy*; *it is important that the dietary management is regularly reviewed with frequent biochemical monitoring. After delivery*, *the Met intake should be reduced to no more than pre-pregnancy levels*.

Thrombosis is a major risk for pregnant women with CBS deficiency. Some women present for the first time with thromboses during pregnancy or the post-partum period (Calvert and Rand 1995). The risk is particularly high for 6 weeks after delivery, while the uterus is involuting: plasma Hcy concentrations are often high at this time despite treatment (Yap et al 2001a, b, c; Levy et al 2002). There are no studies of the optimal timing or form of anticoagulation in CBS deficiency but thrombophilia is considered in many thromboprophylaxis guidelines. All patients should have anticoagulation for the third trimester and continue it for 6 weeks post-partum; women with additional risk factors or a previous thrombosis should usually have anticoagulation throughout pregnancy. Low molecular weight heparin is used most widely but subcutaneous heparin has also been recommended (Calvert and Rand 1995; Yap et al 2001a, b, c). Warfarin has also been used in the post-partum period (Calvert and Rand 1995).

Management of CBS deficiency during pregnancy depends on the phenotype. For example, individuals who have pyridoxine-responsive CBS deficiency should remain on their pyridoxine through pregnancy; there is some weak evidence that this improves outcomes, though this is based on only a few patients (Mudd et al 1985; Levy et al 2002). Adjuvant therapies may be necessary to maintain adequate energy and protein intake and appropriate Hcy levels. Betaine has been used in pregnancy without adverse effects (Yap et al 2001a, b, c; Pierre et al 2006). All pregnant patients should receive folate supplementation (at least 400 micrograms), along with vitamin B_12_ if there is evidence of deficiency.

In pyridoxine unresponsive CBS deficient women, there is an increased Met requirement as the fetus grows. The aim is to increase Met intake while still maintaining good biochemical control (Yap et al 2001a, b, c). The WHO/FAO/UNU recommend additional protein intake in pregnant women (0.5 g/day in trimester 1, 8 g/day in trimester 2 and 25 g/day in trimester 3). Increased L-AA supplements may be required if the increase in natural protein tolerance is insufficient to meet these requirements. It is important that the dietary management is regularly reviewed with frequent biochemical monitoring. Zinc and selenium deficiencies have been found in some women (Pierre et al 2006). Monitoring of fibrinogen as a secondary marker of coagulopathy can be helpful in patients with poor control (Ritchie and Carson 1973; Yap et al 2001a, b, c).

After delivery, the Met intake should be reduced to no more than pre-pregnancy levels (Yap et al 2001a, b, c). However, the WHO/FAO/UNU protein requirements during lactation recommend an additional 20 grams protein/day, falling to 12.5 grams per day after 6 months. Some of this will need to be given as Met-free L-AA supplements, guided by biochemical monitoring.

### Special management issues

#### Management of intercurrent illness (statement #37: grade of recommendation D)

*During intercurrent illness*, *patients should continue their regular treatment* (*such as pyridoxine*, *betaine*, *their L-AA mixture and adequate energy). Dehydration and immobilization should be avoided to reduce the risk of thromboembolic disease*.

Venous thrombosis is the main acute concern and the risk of this should be minimised by avoiding dehydration or immobilisation. Intractable vomiting may necessitate intravenous fluids.

None of the treatments for homocystinuria need to be stopped during intercurrent illnesses. Some increase in Hcy during illness is to be expected due to catabolism and should not be a concern for isolated, short illnesses. More frequent nutritional monitoring may be needed in patients on dietary treatment who have prolonged or recurrent illnesses.

#### Management of surgery and anaesthesia (statement #38: grade of recommendation C-D)

*Surgery and anaesthesia pose an additional risk of thrombosis in CBS deficiency. Biochemical control*, *dietary management and nutrition should be optimised before elective procedures. Hydration should be maintained with intravenous fluids. Standard anti-thrombotic measures such as elastic stockings, pneumatic leg compression systems and early mobilization should be followed during and after surgery. Low molecular weight heparin is recommended in cases of prolonged immobilisation. Nitrous oxide increases Hcy concentrations and should be avoided*.

There are multiple reports of previously diagnosed and undiagnosed patients with thomboses during surgery (Carson et al 1965; Morreels et al 1968; Elkington et al 1973; Harker et al 1974; Grieco 1977; Harker and Scott 1977; Mudd et al 1985; Arbour et al 1988). These can be fatal (Carson et al 1965; Mudd et al 1985), if not aggressively treated. The risk is even higher in arteriography/venography. In a historical cohort of 241 major surgical procedures there were 14 post-operative thromboembolic events with four being fatal (Mudd et al 1985).

Although there have been no clinical trials, there is no question that control should be optimised prior to elective surgery and maintaining hydration is important. One should also consider consulting a haematologist and an anaesthetist before elective procedures. Historically dipyridamole, aspirin and warfarin have been used to prevent thromboembolism (Harker et al 1974; Schulman et al 1978) but low molecular weight heparin prophylaxis is now recommended. Early post-operative mobilisation and inflation compression stockings are also recommended (Lowe et al 1994; Asghar and Ali 2012), as in other states associated with an increased risk of deep venous thrombosis.

Nitrous oxide inactivates cobalamin, thereby inhibiting methionine synthase. Rises in Hcy have been reported following its use during anaesthesia. Nitrous oxide should be avoided in patients with CBS deficiency and, particularly, if there is vitamin B_12_ deficiency, when it can cause subacute combined degeneration of the cord.

#### Precautions for travel (statement #39: grade of recommendation D)

*Standard measures for preventing thrombosis are recommended for travel*.

There are no publications concerning travel and homocystinuria. Guidelines for patients who have had a venous thromboembolism are the same as for patients with thrombophilia—i.e. prophylactic low molecular weight heparin (Guyatt et al 2012). There are no guidelines for patients who have not had thromboembolism, but encouraging mobility and maintaining hydration is appropriate (Biasiutti and Lammle 1994).

### Outcome (statement #40: grade of recommendation C-D)

*Without treatment*, *life expectancy is markedly reduced in pyridoxine-unresponsive patients. The life expectancy of pyridoxine-responsive patients is uncertain due to under-ascertainment in studies of this group. With early life-long adequate treatment, outcomes are generally good, although the very long-term outcomes are not yet known. Late teenagers and young adults are at high risk of non-compliance and complications, including death. Outcomes are determined primarily by pyridoxine responsiveness, adequacy of treatment and age of detection*.

If untreated, the prognosis of pyridoxine-unresponsive CBS deficiency is bleak (Carson et al 1965; Schimke et al 1965; Mudd et al 1985). The consequences of untreated, or partially treated, CBS deficiency include thromboembolic events, mental retardation, ocular and skeletal manifestations. An international study that documented the natural history of 629 untreated CBS patients showed that the risk of complications increases with age, and that pyridoxine-unresponsive patients are more severely affected (Mudd et al 1985). Pyridoxine responsive patients had significantly better mental capacities (n = 107, mean 79) than pyridoxine-unresponsive patients (n = 115, mean 57). The results of this study, however, were subject to ascertainment bias as milder cases, associated with pyridoxine responsiveness, were probably under-represented (Skovby et al 2010). Pyridoxine-responsive patients who present with thromboembolism as adults usually have a normal IQ and no other complications.

Long-term treatment, with good biochemical control, prevents complications from developing but it does not reverse complications already present. Moreover, loss of biochemical control at any age is associated with serious complications, which may be life-threatening. Thus, for pyridoxine unresponsive patients, optimal outcomes require NBS and treatment shortly after birth. No complications were observed in 15 such patients from Ireland, aged up to 25 years, whose lifetime median fHcy was maintained below 11 μmol/L (Yap and Naughten 1998). They all had excellent vision, with full scale IQ ranging from 84-120 (mean 105.8) in 13 of the early treated and compliant patients (Yap et al 2001a, b, c). These patients are now aged up to 43 years and outcomes remain good in those whose plasma fHcy has remained less than 11 μmol/L with only brief rises above this (Crushell E and O’Sullivan S, personal communication). Similar findings were reported in 11 pyridoxine unresponsive, early treated patients from Manchester, aged up to 25 years (Walter et al 1998), with full scale IQ ranging from 84-117 (median 100). The outcome for pyridoxine non-responsive patients diagnosed clinically (and therefore later) was poor with a median IQ of 58 (n = 2).

### Quality of life (statement #41: grade of recommendation D)

*There are no data regarding the quality of life from the patient*’*s or parent*’*s point of view and this area requires further study*.

A systematic search of the PubMed and Cochrane databases using the terms “quality of life” combined with “CBS deficiency” or “homocystinuria” identified no publication addressing this. There are also very few publications concerning employment or independence. Bhat documented the social outcome in treated individuals attending one metabolic centre in the UK (Bhat et al 2005). Only five out of 33 were detected on newborn screening. Marginally higher employment rates were found in the pyridoxine-responsive group, and more of the pyridoxine responsive patients were in long term relationships. This was, however, subject to ascertainment bias, as the questionnaire was completed by patients.

Biochemical and physical outcome parameters are generally used for patients with inborn errors of metabolism but quality of life (QoL) should also be assessed (Zeltner et al 2014). Psychosocial factors such as coping strategies predict health-related QoL in patients with chronic disease more precisely than physical parameters (Cohen and Biesecker 2010). Moreover, QoL assessments are essential for an overall estimation of treatment success (Simon et al 2008).

For patients with metabolic diseases, major concerns include dietary restrictions, blood sampling, social stigmatization and exclusion (Zeltner et al 2016). Delay in diagnosis is a major concern for many families as accurate diagnosis is the first step to improving the care for those living with a rare disease (eurordis.org/IMG/pdf/voice_12000_patients/EURORDISCARE.FULLBOOKKr.pdf) (retrieved March 2015).

The impact on families should not be underestimated. The parents of a child with a metabolic disorder are likely to have lower health related QoL than parents of children with other chronic diseases (Hatzmann et al 2009). A study of Australian families caring for children with genetic metabolic disorders found they were adversely affected by delays in diagnosis, poor access to peer support groups and lack of psychological support (Anderson et al 2013).

Research is needed to identify which strategies are most effective in improving QoL for patients with homocystinuria.

## Conclusions

These guidelines are based on the best data currently available concerning the diagnosis, biochemistry, complications and management of CBS deficiency. Unfortunately, many of our recommendations are grade D, due to a lack of high grade published evidence. The guidelines should be used to identify the areas in which data are lacking and to facilitate the design of international collaborative studies, using the same diagnostic measures and therapies. We envisage revising the guidelines in 5 years to incorporate data from such studies and other advances. The ultimate goal of these guidelines is to improve the lives and health of patients with pyridoxine-responsive and -unresponsive CBS deficiency now and in the future.

## Abbreviations

AA: Amino acid
CBS: Cystathionine beta-synthase
D: Dioptres
DBS: Dry blood spots
DEXA: Dual energy X-ray absorptiometry
EEG: Electroencephalography
E-HOD: European network and registry for homocystinurias and methylation defects
GDG: Guideline Development Group
Hcy: Homocysteine
fHcy: Free homocystine
tHcy: Total homocysteine
L-AA: L-amino acid
Met: Methionine
MRI: Magnetic resonance imaging
NBS: Newborn screening
PKU: Phenylketonuria
QoL: Quality of life
RCT: Randomised clinical trial
SAM: S-adenosylmethionine
SAH: S-adenosylhomocysteine
SIGN: Scottish Intercollegiate Guideline Network
WHO/FAO/UNU: World Health Organization/Food and Agriculture Organization of the United Nations/United Nations University
